# Cell non-autonomous regulation of cerebrovascular aging processes by the somatotropic axis

**DOI:** 10.3389/fendo.2023.1087053

**Published:** 2023-01-23

**Authors:** Marisa A. Bickel, Boglarka Csik, Rafal Gulej, Anna Ungvari, Adam Nyul-Toth, Shannon M. Conley

**Affiliations:** ^1^ Department of Cell Biology, University of Oklahoma Health Sciences Center, Oklahoma City, OK, United States; ^2^ Vascular Cognitive Impairment, Neurodegeneration and Healthy Brain Aging Program, Department of Neurosurgery, University of Oklahoma Health Sciences Center, Oklahoma City, OK, United States; ^3^ Oklahoma Center for Geroscience and Healthy Brain Aging, University of Oklahoma Health Sciences Center, Oklahoma City, OK, United States; ^4^ International Training Program in Geroscience, Department of Public Health, Semmelweis University, Budapest, Hungary; ^5^ Institute of Biophysics, Biological Research Centre, Eötvös Lorand Research Network (ELKH), Szeged, Hungary

**Keywords:** dementia, microbleed, hormonal, humoral, ageing

## Abstract

Age-related cerebrovascular pathologies, ranging from cerebromicrovascular functional and structural alterations to large vessel atherosclerosis, promote the genesis of vascular cognitive impairment and dementia (VCID) and exacerbate Alzheimer’s disease. Recent advances in geroscience, including results from studies on heterochronic parabiosis models, reinforce the hypothesis that cell non-autonomous mechanisms play a key role in regulating cerebrovascular aging processes. Growth hormone (GH) and insulin-like growth factor 1 (IGF-1) exert multifaceted vasoprotective effects and production of both hormones is significantly reduced in aging. This brief overview focuses on the role of age-related GH/IGF-1 deficiency in the development of cerebrovascular pathologies and VCID. It explores the mechanistic links among alterations in the somatotropic axis, specific macrovascular and microvascular pathologies (including capillary rarefaction, microhemorrhages, impaired endothelial regulation of cerebral blood flow, disruption of the blood brain barrier, decreased neurovascular coupling, and atherogenesis) and cognitive impairment. Improved understanding of cell non-autonomous mechanisms of vascular aging is crucial to identify targets for intervention to promote cerebrovascular and brain health in older adults.

## 1 Introduction

Age-related cognitive impairment and dementia are major public health challenges in the rapidly aging societies of the developed world. In older adults, cognitive impairment of vascular etiology [vascular cognitive impairment and dementia or VCID (1)] is the second most common cause of clinically diagnosed dementia after Alzheimer’s disease (AD) (2–4). VCID is also one of the most frequent causes of loss of independence and increased morbidity in older adults (1). Vascular pathologies are also a critical component of AD, and vascular dysfunction is one of the earliest pathologies to appear in patients with mild cognitive impairment, many of whom go on to develop dementia (5–8).

In addition to large vessel disease, age-related VCID is associated with a wide variety of microvascular pathologies (9–17). Microvascular contributions to cognitive decline and dementia include microvascular rarefaction (18–21), impaired endothelial regulation of cerebral blood flow (5, 22–30), disruption of the blood brain barrier (BBB) (19, 31–36), decreased neurovascular coupling (NVC) (37–41), cerebral microhemorrhages (CMH) (42–44), lacunar infarcts (45–49), increased pulsatility (50–53) and small vessel disease-related white matter damage (54–57), and amyloid pathologies (58–62). Critically, the severity of age-related increases in microvascular pathological alterations predict cognitive decline in aging (44, 63, 64), leading to great interest in understanding the associated cellular and molecular mechanisms. Additionally, age-related microvascular pathologies also exacerbate severity of ischemic brain injury (65–67).

There is growing preclinical evidence that interventions that promote cerebromicrovascular health and rejuvenation (68–71) have beneficial effects on cognitive health in aging. Understanding the mechanisms implicated in age-related impairment of the cerebral circulation is essential for identification of novel targets for translationally relevant interventions and development of innovative therapies to promote healthy cerebrovascular and brain aging. In this review, the effect of aging on a critical endocrine pathway, the somatotropic axis, and its role in regulating the functional and structural integrity of the cerebral circulation is considered in terms of potential mechanisms involved in age-related dysregulation of cerebral blood flow and increased susceptibility to microvascular damage.

## 2 Regulation of aging processes by the GH/IGF-1 axis

The neuroendocrine hypothesis of aging posits that changes in endocrine output of the hypothalamic-pituitary axis regulate the process of organismal aging in a cell non-autonomous manner (72). There is particularly strong evidence that among the related endocrine factors, the somatotropic axis, including growth hormone (GH) and insulin-like growth factor-1 (IGF-1), exerts a central role in regulation of cellular processes involved in aging.

**Figure 1:**
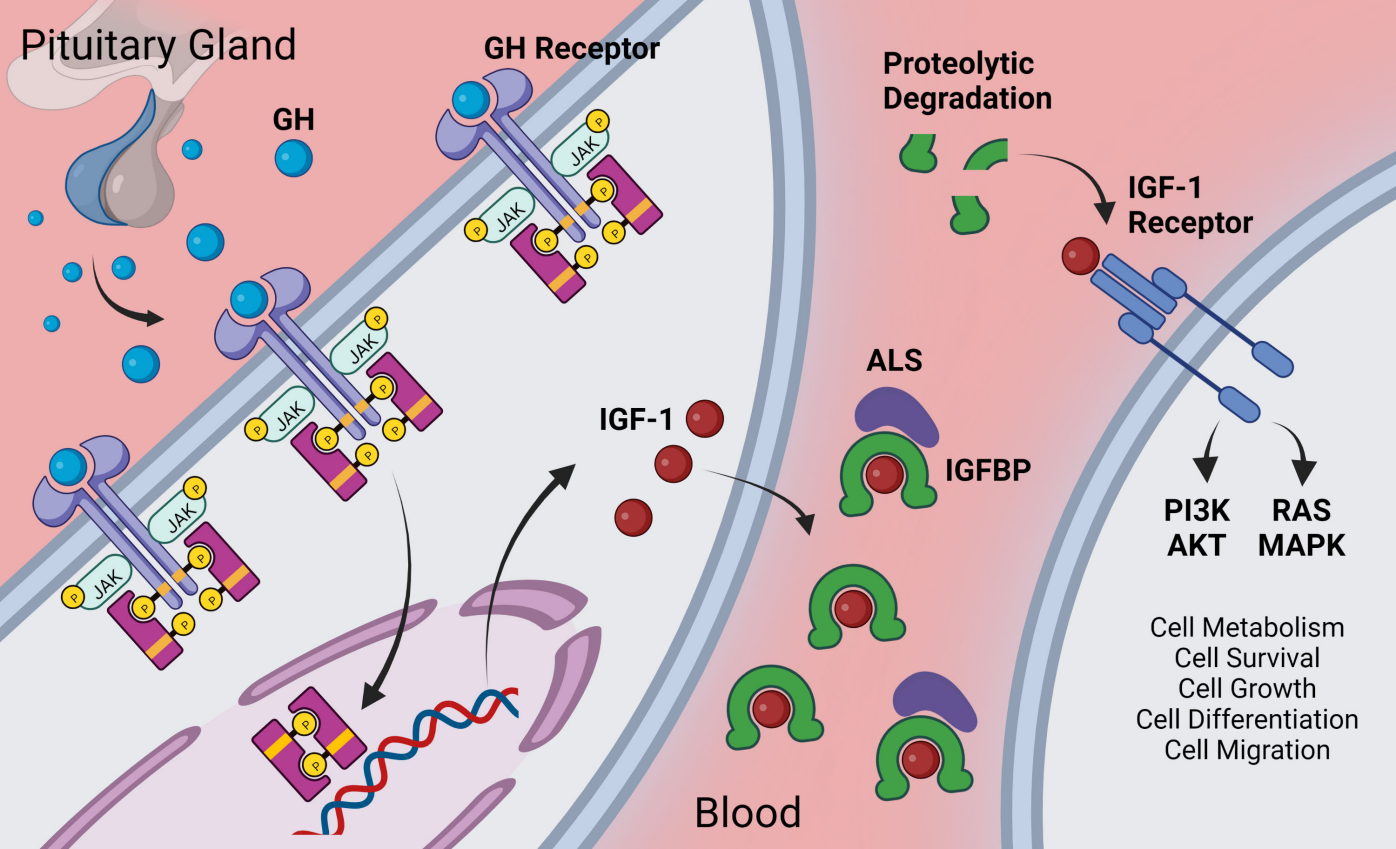
Overview of GH/IGF-1 signaling axis. Growth hormone is secreted from the anterior pituitary gland. It acts *via* the GH receptor on target cells *via* Janus kinase (JAK)-signal transducer and activator of transcription (STAT) –signaling to promote expression of various genes including IGF-1. Target cells include hepatocytes (for endocrine/circulating IGF-1) as well as other cells throughout the body that locally secrete IGF-1. Extracellular IGF-1 is bound to IGF-1 binding proteins (IGFBP) either in a two-part complex or in a three-part complex with the glycoprotein acid labile subunit (ALS). IGF-1 has higher affinity for IGFBPs than for the IGF-1 receptor, and IGF-1 is released from the IGFBP upon proteolytic cleavage of the IGFBP [for more on the complex role of IGFBPs, see (85)]. Freed IGF-1 interacts with the IGF-1 receptor where it signals, largely through the PI3K/AKT and the RAS/MAPK cascades to regulate many pro-survival cellular pathways. Created with BioRender.com.

IGF-1 is an evolutionarily highly conserved pleiotropic anabolic hormone and growth factor (73–84). It exhibits high sequence similarity to insulin and is a member of a complex intercellular signaling system. IGF-1 is secreted by the liver as a result of stimulation by GH and is also produced locally by a number of cell types where it acts in a paracrine and autocrine manner (including cardiovascular cells, astrocytes and neurons). The GH/IGF-1 axis also includes cell-surface receptors (IGF1R and IGF2R) and a family of high-affinity IGF-binding proteins (IGFBP1 to IGFBP7), as well as associated IGFBP degrading proteases (see overview in **Figure 1**). GH is secreted from the anterior pituitary gland in response to GH releasing hormone, and acts on the liver and other tissues to promote the secretion of IGF-1 (86). The GH/IGF-1 axis is essential for proper growth and development (87–89) and confers multifacteed pro-survival, anabolic and cellular protective effects. Levels of GH decrease by ~14%/decade after approximately the third decade of life (90, 91), and as a result levels of circulating IGF-1 also significantly decrease with age (92–95).

The role of decreased GH and IGF-1 in aging has been extensively studied. Age-related GH/IGF-1 deficiency has been causally linked to the genesis of aging phenotypes in various organ systems, including the cardiovascular system, musculoskeletal system and the central nervous system (95–105). Patients with decreased GH/IGF-1 have an increased risk of VCID and other forms cardiovascular and cerebrovascular disease (94, 106–108), including gait and cognitive impairment (109–111), as well as diabetes mellitus (106). Older adults with low circulating IGF-1 levels have 39% higher risk of cardiovascular mortality (112). Animal models of circulating IGF-1 deficiency serve as models of accelerated aging (18, 21, 93, 113–120), mimicking many age-related cerebrovascular pathologies.

Many cytoprotective and anti-aging effects of GH and IGF-1 have been described, and the protective role of the GH/IGF-1 axis in regulation of the development of age-related diseases (e.g. *via* modulation of metabolism, protein synthesis, glucose metabolism, cellular proliferation and differentiation) is well-supported by the literature (10, 93, 97, 98, 102, 103, 105, 115, 116, 119, 121–134). However, the role of GH/IGF-1 in the regulation of lifespan is admittedly complex and highly controversial. There is strong experimental evidence suggesting that disruption of GH/IGF-1 signaling (or of their orthologs in lower organisms) is often associated with lifespan extension both in invertebrate model organisms (*C. elegans*, *D. melanogaster*) and laboratory rodents (135, 136), including Ames and Snell dwarf mice (135–141), the ‘little mouse’ *(Ghrhr^lit/lit^)*, mice null for either GH receptor/binding protein *(GHR/BP^-/-^)* or p66(shc) (*p66(shc^-/-^)*), GHRH and GHR double-knockout mice (142), mice heterozygous for the IGF-I receptor (*Igf1r^+/-^*), and fat-specific insulin receptor knockout mice (143–152). Interestingly, GH receptor knockout (GHRKO) mice (153), which also have low IGF-1 levels, hold the Methuselah prize for the world’s longest-lived laboratory mouse. There have been several attempts to reconcile these two, apparently contradicting, aspects of the “GH/IGF-1 paradox of aging” (98, 154). A key observation is that dwarfism in murine (Ames and Snell dwarf mice and the ‘little mouse’) and rat [spontaneous dwarf rat (155)] models caused by early-onset disruption of the GH/IGF-1 axis is associated with longevity [a notable exception being the Lewis dwarf rat (156)]. Importantly, the remarkable life-span extension of hypopituitary Ames dwarf mice was shown to depend on low levels of GH in a relatively short peripubertal time-window (149, 154). These data raised the possibility that in murine models, GH/IGF-1 regulates lifespan primarily through developmental programming of aging (154). Despite the significant progress made in the field of geroscience in the past two decades, the impact of disruption of the GH/IGF-1 axis on human lifespan and longevity is vigorously debated (98, 157–163). Overall, the available epidemiological evidence has suggested that neither early-life nor late-life disruption of GH/IGF-1 signaling in humans extends lifespan (98). While the literature on the role of the GH/IGF-1 axis in modulating aging processes, lifespan and the development of specific age-related diseases is large, here we focus on links between the GH/IGF-1 axis and manifestations of cerebrovascular aging.

## 3 Role of GH/IGF-1 in cerebrovascular remodeling

Blood vessels undergo constant functional and structural remodeling to respond to changing tissue demands, metabolic conditions, and injury repair (164, 165). Structural remodeling consists of proangiogenic processes, vascular quiescence, and vascular regression. Angiogenesis provides increased blood supply in a long-term manner to tissues with high metabolic demand, for example exercise-mediated angiogenesis and uterine artery adaptation during pregnancy. During vascular quiescence, vascular cells stop proliferating and undergo further maturation, specialization, and stabilization. During this phase, brain endothelial cells form tight and adherens junctions, providing a physical barrier in vessels. Lastly, blood vessels can undergo vascular regression including vascular involution, in which an extended vascular network regresses, or vascular pruning in which single vessels regress. These processes are crucial for fine-tuning the hierarchical organization of blood vessels, providing highly organized networks of arteries, capillaries, and veins. The mechanisms and regulation of the processes involved in vascular remodeling have been extensively reviewed by Ouarné et al. (164). Dysregulation of vascular remodeling can lead to and exacerbate several vascular and non-vascular pathologies. For example, AD is associated with excessive microvascular pruning, vasoconstriction, and brain hypoperfusion (164). Aging is also associated with pathological vascular remodeling, predominantly characterized by changes in wall rigidity, increased fragility, and vascular rarefaction (72).

### 3.1 The ECM in IGF-1 mediated vascular remodeling

The extracellular matrix (ECM) plays a critical role in vascular remodeling. In addition to providing structural support to tissues, the ECM is involved in transducing biochemical and biomechanical signals, modulating adhesion of adjacent cells, and regulating differentiation, migration, and stability. The ECM constantly undergoes a qualitative and quantitative remodeling process mediated by specific enzymes such as matrix metalloproteinases (MMPs), a disintegrin and metalloproteinases (ADAMs), and meprins (166), and altered ECM remodeling contributes to pathologies such as AD and cancers (72, 167). Aging is also characterized by dysregulation of vascular ECM remodeling (72, 168, 169). With age, ECM biosynthesis decreases, and there are alterations in cell-matrix attachments, biomechanical signaling, and balance between proteases and their inhibitors. These age-mediated changes in the ECM contribute to vascular pathologies such as arterial stiffening, loss of BBB integrity, vascular fragility, and the development of CMH (72, 169, 170).

**Figure 2:**
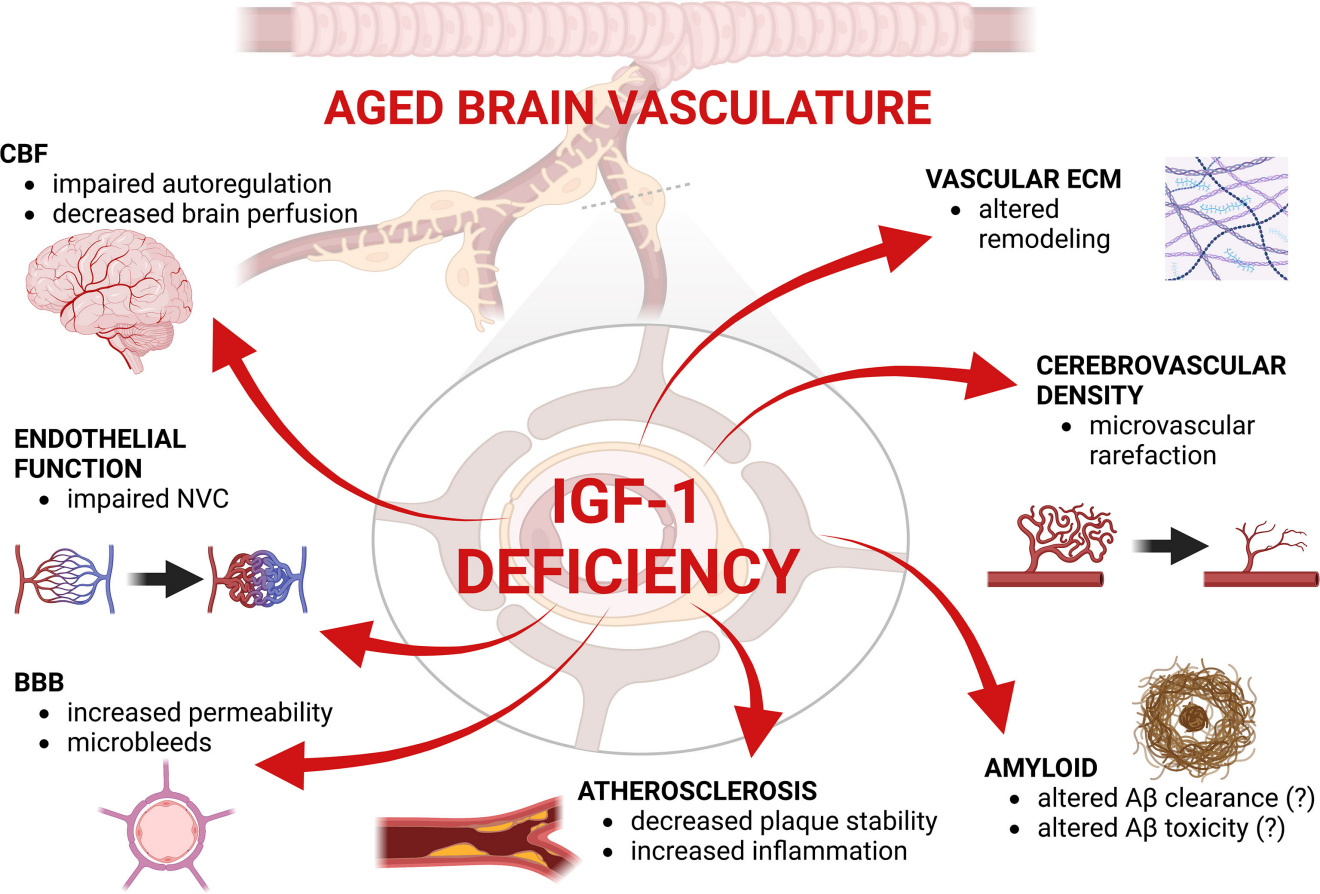
Summary figure highlighting the effects of IGF-1 deficiency in the aged brain vasculature. CBF, Cerebral blood flow; ECM, extracellular matrix; BB, blood-brain barrier; NVC, neurovascular coupling. Created with BioRender.com.

IGF-1 deficiency has been shown to lead to defects in vascular remodeling (**Figure 2**). One of the most widely-used models to study IGF-1 deficiency in aging is an adult-onset circulating IGF-1 knockdown model in which liver-specific IGF-1 knockdown is induced post-development (~3-6 months of age) *via* injection of AAV-TBG-Cre. When this model is exposed to hypertension as a cerebrovascular challenge, IGF-1 deficiency increased MMP activation and oxidative stress (93), increased vessel rigidity, promoted medial atrophy, and decreased vessel elasticity (114). Hypertension is normally associated with protective ECM remodeling (largely regulated by vascular smooth muscle cells [VSMCs]), including upregulation of ECM cross-linking genes (e.g. *lox, loxl1, loxl4*), elastin and elastin-associated genes (e.g. *eln, fbn1*), and some collagens (e.g. *Col1a1, Col3a1, Col 8a1*). However, this remodeling response was blunted or abolished in mice with circulating IGF-1 deficiency (114). Support for a role of IGF-1 in the maintenance of vascular stability through ECM proteins comes from the observation that across seven different mouse strains, aortic collagen levels were positively correlated with serum IGF-1 levels (171).

### 3.2 Role of GH/IGF-1 in regulation of cerebral capillary density

Cerebrovascular complexity and high vessel density are essential for healthy brain function, but decrease with age, manifesting as microvascular rarefaction in humans and animal models (172–175). Age-related microvascular rarefaction has been causally linked to cognitive decline (176–178) and other organ-specific manifestations of organismal aging (179–182).

A role for circulating/endocrine factors in age-related microvascular loss is highlighted by recent work showing that exposure to young blood *via* heterochronic parabiosis reversed age-related microvascular rarefaction and hypoperfusion (183–185). Analysis of upstream transcriptional regulators identified IGF-1 receptor signaling as one of the candidates responsible for this rejuvenation. IGF-1 is a potent pro-survival factor in both endothelial cells and VSMCs, and microRNA-mediated knockdown of IGF-1 receptor in endothelial cells can induce apoptosis *in vitro* (186). Consistent with this, decreased cerebrovascular density is observed in IGF-1 deficient models. Animals with decreased circulating IGF-1 exhibit hippocampal microvascular rarefaction (21, 178), and circulating IGF-1 has been shown to be essential for exercise induced increases in cerebral vascular density (**Figure 2**) (18, 187). Treatment with IGF-1 has also been shown to promote cerebrovascular angiogenesis and increase vessel density, for example in post-stroke models (188, 189) and in the normal adult mouse brain (187). Increased IGF-1 levels associated with high circulating GH also result in increased retinal microvascular density (190, 191). The dynamic balance between the processes of angiogenesis and capillary regression is essential for maintenance of the optimal network architecture of the cerebromicrovasculature. Aging is associated with a progressive deterioration of microvascular homeostasis, at least in part due to age-related impairment of angiogenic processes (173, 192–196). IGF-1 is known to confer potent and multifaceted pro-angiogenic effects, whereas IGF-1 deficiency impairs multiple aspects of angiogenesis (197–201). The pro-aging effects of endocrine GH/IGF-1 deficiency may be exacerbated by an age-related decline in other vasculoprotective growth factors, including pituitary adenylate cyclase-activating polypeptide (PACAP) (202) and vascular endothelial growth factor (VEGF) (203–205) and endothelial resistance to the effects of pro-angiogenic stimuli (206).

Because of the tight relationship between GH/IGF-1, some studies have supplemented animals with GH in order to induce endogenous production of IGF-1. In aged rats, supplementation with GH not only increased systemic levels of IGF-1 but also increased the density of microvessels within the top layer of the cerebral cortex (178). Combined these data highlight a clear role for IGF-1 deficiency in microvascular rarefaction in the brain.

### 3.3 Role of GH/IGF-1 in remodelling of larger vessels

In addition to microvascular rarefaction, aging is also associated with defects in remodeling in larger vessels. In parabiosis studies, the rejuvenating effects of young blood were also observed in macrovasculature. Aortas from aged mice exposed to young blood showed improved functional remodeling manifested as restored endothelium-mediated vasorelaxation and decreased oxidative stress. Additionally, upstream regulator analysis (via IPA) of the transcriptome of rejuvenated aortas suggested that rejuvenation was associated with activation of the IGF-1 pathway (185). On the other hand, young aortas exposed to aged blood also showed significant transcriptomic changes. Gene ontology analysis revealed that exposure to aged systemic factors upregulated genes associated with pathologic vascular remodeling. IPA upstream analysis showed that pro-geronic effects of aged blood might include inhibition of pathways mediated by IGF-1, serum response factor, and vascular endothelial growth factor (VEGF-A) (184). Recent human studies support the hypothesis that cell non-autonomous mechanisms contribute to age-mediated changes in vascular remodeling. Yu et al. identified a protein signature in the urine of older individuals that is associated with vascular remodeling (207). Human studies have also highlighted a role for the somatotropic axis in ECM regulation in the vasculature. Patients with uncontrolled acromegaly, a disease caused by hypersecretion of GH (and characterized by consequent increase in IGF-1 levels), exhibit baseline increases in vessel wall thickness and increased wall-to-lumen ratio in retinal arterioles compared to controls (191). This is similar to what has been observed in the GH overexpressing transgenic mouse, which exhibits increased medial layer area in the aorta and mesenteric vessels (208). This GH/IGF-1 mediated increased layer thickness is likely due to both altered ECM deposition and cell proliferation. Indeed VSMC proliferation has been reported in the aorta after perfusion with IGF-1 in rat diabetic aortic catheterization model (209). These structural changes have functional correlates, and it has been shown that noradrenaline-induced VSMC-mediated aorta contraction is increased in mice pre-treated with IGF-1 (210).

Combined these findings suggest that IGF-1 deficiency is associated with microvascular rarefaction and impaired vascular remodeling in response to stressors such as hypertension. In contrast, overexpression of IGF-1/GH can lead to excess vascular wall hypertrophy, highlighting a central role for the somatotropic axis in the regulation of vascular wall growth. Much of this regulation is tied to IGF-1 mediated changes in ECM gene expression, in particular regulation of elastin and elastin-associated genes, which is consistent with the importance of maintaining appropriate responses to mechanical stress in the vasculature.

## 4 Role of GH/IGF-1 in regulation of cerebrovascular function

### 4.1 GH/IGF-1 in regulation of cerebral blood flow and autoregulatory reactivity

The limited energy storage and high metabolic rate of the brain demands a constant flow of blood to deliver oxygen and nutrients and remove cellular, metabolic, or toxic by-products. There are several overlapping mechanisms which regulate cerebral blood flow (CBF) to maintain the baseline flow as well as mediate activity-dependent adjustment in flow to increased oxygen and nutrient demand (211).

To help maintain constant intravascular blood pressure in the brain in the face of systemic changes in blood pressure, cerebral vessels have a myogenic autoregulatory response system, where the arteries and arterioles in the pial and parenchymal circulation respond to changes in intraluminal pressure with changes in vascular tone and diameter (211). Healthy young animals exhibit structural (increased wall thickness) and functional (increased myogenic tone) adaptation of the proximal arterial branches of the cerebrovascular tree in response to permanent increases in blood pressure, thereby maintaining normal pressure and blood flow in the thin-walled, injury-prone downstream portion of the microcirculation (212). There is growing evidence that aging is associated with functional (myogenic autoregulatory dysfunction) and structural maladaptation to increased blood pressure in the cerebral circulation (213, 214), which has been causally linked to the increased susceptibility to the development of microhemorrhages and BBB disruption. Age-related arterial stiffening results in an increased pulse pressure which causes increased pulsatility in CBF in elderly individuals (215). Studies show that in aged mice, the myogenic response to static increases in pressure is intact in isolated middle cerebral arteries, but is significantly impaired in response to increases in pulsatile pressure (213, 214), while responses in aged parenchymal arterioles were impaired in response to static pressure (214). Functional maladaptation of aged cerebral arteries to hypertension is partly due to the dysregulation of the transient receptor potential canonical channel 6 (TRPC6), which is a non-selective cation channel from the transient receptor potential (TRP) ion channel superfamily. In young animals, increased blood pressure activates TRPC6, which is sensitive to wall stretch (due to increased intraluminal pressure), thus leading to the depolarization of the VSMC plasma membrane, opening of voltage-gated Ca^2+^ channels, increasing intracellular Ca^2+^ concentration, and consequent constriction of VSMCs (216). Impaired autoregulatory protection in the brain of hypertensive aged mice aggravates cerebromicrovascular injury and neuroinflammation (217) by allowing high pressure to penetrate the distal portion of the cerebral microcirculation.

Reductions in circulating IGF-1 may contribute to this age-related loss of adaptive ability (115). Circulating IGF-1-deficient mice exhibit significant impairment of cerebrovascular autoregulation compared to control mice (115) (**Figure 2**), mimicking the phenotype seen in aging (217). Autoregulatory impairment was most pronounced in hypertensive IGF-1 deficient mice, who also failed to exhibit the protective increase in myogenic tone and protective increases in TRPC6 channel expression that accompany hypertension in control mice (115). This functional maladaptation of cerebral arteries to hypertension in IGF-1 deficient animals correlates with the structural maladaptations described in section 3, in particular the significant reduction in hypertension-induced adaptive hypertrophy and decreased elasticity in the medial layer of vessels in IGF-1 deficient animals (114). Studies on isolated aorta preparations also suggest that IGF-1 regulates contractile function of vascular smooth muscle cells (210).

Collectively, impaired autoregulatory function and impaired protective vessel remodeling can lead to damaging increases in pressure in the cerebral microvasculature. High intraluminal pressure is a key stimulus for increased vascular production of reactive oxygen species (ROS) (218). Previous studies showed that in aging, increased oxidative stress led to matrix metalloprotease (MMP) activation (219) thereby compromising the structural integrity of the cerebral microvasculature. Similarly, hypertensive IGF-1‐deficient mice exhibit increased vascular ROS and increased vascular MMP activity compared to control mice (93).

IGF-1 may also play a role in regulating baseline CBF. Magnetic resonance imaging studies have shown that baseline mean blood flow velocity in the middle cerebral artery is decreased in aged vs. young cohorts, and that flow is significantly correlated with serum IGF-1 levels (94). Overall, IGF-1 has a central role in regulation of CBF by contributing to pressure- and flow-dependent responses of cerebral arteries, plays a role in structural adaptation to hypertension, and contributes to adaptive ECM changes and in ECM-related gene expression. IGF-1 deficiency dysregulates the myogenic response to high blood pressure, it impairs the hypertension-induced adaptive media hypertrophy and leads to dysregulation of ECM remodeling contributing to increased fragility of intracerebral arterioles and exacerbating cerebromicrovascular injury and neuroinflammation mimicking the aging phenotype.

### 4.2 Role of GH/IGF-1 in regulation of microvascular endothelial function and neurovascular coupling responses

Neurovascular coupling (NVC) is the ability of the neurovascular unit (NVU) to increase local blood flow based on neuronal activity/energy requirements and is critical for maintaining proper brain function. The NVU is comprised of neurons, glial cells, and the vascular subunit (brain endothelial cells, pericytes and the surrounding basement membrane) (220). NVC is the result of a tightly controlled interaction between activated neurons and astrocytes which leads to the release of vasodilator metabolites from the astrocyte end-feet and microvascular endothelial cells. These metabolites include nitric oxide (NO), potassium, adenosine, epoxyeicosatrienoic acids and prostaglandins and collectively elicit vasodilation in arterioles (221, 222).

The cellular mechanisms by which aging impairs neurovascular coupling responses primarily involve a significant reduction in endothelial production/release of NO (223–225). Neurovascular dysfunction compromises adjustment of cerebral blood flow to meet the needs of active brain regions, impairing energy and oxygen delivery to the firing neurons and hindering washout of toxic metabolic by-products (226). Cells of the neurovascular unit (including neurons, astrocytes, and endothelial cells) abundantly express the IGF-1 receptor, as IGF-1 signaling can also play a role in endothelial mediated vasodilation (227, 228). IGF-1 mediated activation of the phosphatidylinositol-3 kinase (PI3-K) pathway in endothelial cells can lead to production of NO by nitric oxide synthase, leading to paracrine signaling on VSMCs, resulting in VSMC relaxation, and subsequent vessel dilation (229, 230).

Laser doppler flowmetry has been routinely used in preclinical models to evaluate NVC. CBF is measured in the somatosensory cortex before and after whisker stimulation, and the increased CBF following stimulation is reflective of NVC response. Stimulation-induced increases in CBF were much lower in circulating IGF-1 deficient mice compared to controls (113, 116). This impairment of NVC in IGF-1 deficiency supports a protective role for IGF-1 in vascular function. Subsequent mechanistic work demonstrated that both endothelium-mediated and astrocyte-dependent NVC responses were reduced in IGF-1-deficient mice, mimicking the aged human condition (231, 232). To help further elucidate the cellular contributions to IGF-1-mediated regulation of NVC, various studies have either over-expressed or knocked out the IGF-1 receptor in specific cell types. Mice overexpressing human IGF-1 receptor in the endothelium were shown to exhibit unaltered vasorelaxation to endothelium-dependent vasodilators (233). However, disruption of endogenous mouse IGF-1 receptor signaling specifically in endothelial cells (VE-Cadherin-*CreERT2/Igf1r^f/f^*) or astrocytes (GFAP-*CreERT2/Igf1r^f/f^*) significantly impaired NVC responses (10, 234). These effects in part are mediated by decreased NO bioavailability due to increased production of ROS, analogous to the effects of circulating IGF-1 deficiency and aging.

IGF-1 also plays a significant role in blood flow changes in response to other types of stimuli such as physical activity. Exercise-mediated neuronal activity elicits changes in cerebral blood flow through both NVC and other regulatory mechanisms. These changes are part of the anti-aging effects of exercise on cerebrovascular and neuronal plasticity. However, these positive effects of exercise were abolished in the circulating IGF-1 knockdown model (235). IGF-1 is also essential for downstream results of NVC in the brain. For example, NVC is an essential component of activity-dependent neurogenesis in the hippocampus (236). However, when IGF-1 signaling in the brain is blocked, activity-dependent but not baseline neurogenesis is eliminated (236).

These preclinical studies have clinical correlates. NVC decreases in aging humans, contributing to VCID (94). In addition, recent work in aged and young study participants show that decreased serum IGF-1 levels are a significant predictor of impaired NVC responses (94). Combined, these findings highlight an essential role for IGF-1 in NVC during aging (105).

### 4.3 GH/IGF-1 in regulation of blood-brain barrier integrity and the development of cerebral microhemorrhages

The BBB is a functional part of the NVU and is critical for the protection of neurons, maintenance of homeostasis, and the integrity of the brain itself (19, 237, 238). BBB dysfunction is one of the hallmarks of the aging brain, in both humans and animal models (19, 33, 34, 239–241). The BBB comprises brain capillary endothelial cells with support from pericytes embedded in the basement membrane and astrocytes. Tight junctions between endothelial cells are a main component of this barrier, leading to the requirement of facilitated transport for nutrient and waste exchange through the capillary endothelial cells. This highly regulated process allows the brain to be an immune-privileged organ giving the BBB an important role as a regulator of neuroinflammation and lymphocyte migration (237, 242–244). Early evidence to support the idea of a multicellular barrier came from work showing that cultured astrocytes induced tighter junctions between endothelial cells (245). When degradation of the BBB begins (for example in inflammaging), small molecules such as cytokines can leak into the surrounding brain tissue leading to subsequent inflammation (19, 243, 244, 246).

GH has a protective role in establishment and maintenance of the BBB during development and in the neonatal brain, especially in models of hypoxia-induced injury (247–249). Recombinant human GH (rhGH) has been shown to have a protective effect in a mouse model of neonatal hypoxic brain injury. Specifically, while hypoxia significantly reduced occludin-positive cortical endothelial cells (a measure of BBB junctional integrity), their frequency was increased in the cortex in response to rhGH (248, 249).

IGF-1 also plays an important role in the maintenance of the BBB. Mice with circulating IGF-1 deficiency have increased BBB permeability (115). This disruption of the BBB can lead to hemorrhaging, neuroinflammation, and neuronal loss (250). IGF-1 also has a well-known protective role in neuroinflammatory processes (251) and has a protective effect on the BBB in other relevant models such as stroke (252–254). IGF-1 supplementation induced Akt activation, reduced blood-brain barrier permeability at 4h poststroke, and suppressed cytokine expression including TNF-α, IL-6, and IL-10 (252). Based on these data, cellular components of the blood-brain barrier may serve as targets of IGF-1 in the aging brain, and IGF-1 supplementation in aged animals and patients may be a useful post-stroke treatment. Recombinant human IGF-1 (rhIGF-1) was able to increase the expression of tight junction proteins (e.g. claudin 5 and occludin) and partially restore BBB integrity in mice with intracerebral hemorrhages (ICHs) (255). Animals treated with rhIGF-1 also showed improved performance on cognitive tests and decreased brain water content compared to ICH mice without rhIGF-1.

In contrast, there is some evidence that a more cautious approach is needed in the application of IGF-1 in the developing brain. At a low dose, IGF-1 delivered intraventricularly significantly reduced lipopolysaccharide (LPS)-induced negative effects such as loss of pre-oligodendrocytes and myelin and in a model of periventricular leukomalacia (a form of brain damage in premature infants) without altering IL-1β expression and microglia/astrocytes activation in the developing brain (256). On the other hand, this low dose of IGF-1 increased LPS-induced BBB permeability, increased polymorphonuclear cell recruitment, and caused ICHs. At higher doses, IGF-1 treatment with LPS highly enhanced mortality of the animals (256).

Penetration of increased pulsatile pressure and pressure surges (e.g. Valsalva maneuver) into the distal, vulnerable part of the cerebral microcirculation can result in rupture of small vessels and genesis of cerebral microhemorrhages (CMHs). CMH are increasingly recognized in T2* and SWI MRI sequences in the majority of older adults. Both preclinical and clinical studies show that advanced age significantly increases the prevalence of CMHs, which contribute to the development of VCID (42–44, 63, 64, 219, 257, 258). CMHs are thought to arise due to a combination of age-associated factors that lead to increased microvascular fragility including: 1) structural defects such as impaired hypertension-induced adaptive changes in the ECM and impaired protective hypertrophy in the medial layer, 2) functional defects such as impaired myogenic autoregulation, and 3) age-related cellular and molecular changes such as increased oxidative stress and MMP activation (93, 114, 115). These age-associated factors are significantly affected by IGF-1 deficiency, and IGF-1 deficiency in mouse models of accelerated aging significantly exacerbates the development of CMHs (93, 113).

## 5 Role of GH/IGF-1 in regulation of amyloid pathologies

Amyloid pathologies (amyloidoses) are a heterogeneous group of diseases characterized by the accumulation of plaques and fibrils made of misfolded proteins. These are formed as a consequence of excessive protein aggregation and/or impairments in the quality control systems responsible for their clearance. Amyloidoses are classified as either systemic or localized. AD is the most commonly diagnosed localized amyloidosis of the central nervous system. A major cause of AD is the aggregation of toxic amyloid-β (Aβ) peptides (259), which are formed when an amyloid precursor protein (APP) is abnormally cleaved. Under physiological conditions, APP is sequentially cleaved by α- and γ-secretases, producing nonamyloidogenic peptides which are essential for neuronal homeostasis. In pathophysiological conditions associated with AD, APP is cleaved by β-secretase and subsequently γ-secretase, generating amyloidogenic Aβ monomers: Aβ_1-40_ and Aβ_1-42_ (260, 261). Decades of studies in AD provide growing evidence that the accumulation of β-amyloid aggregates plays a central role in the pathogenesis of AD, however, mechanisms underlying genesis and regulation of this molecular hallmark of AD remain incompletely understood.

Results from research groups studying GH/IGF-1 signaling in AD are inconsistent, making drawing meaningful conclusions difficult (262). Meta-analysis focused on IGF-1 levels in AD patients revealed that individuals with dementia or AD had lower levels of circulating IGF-1 than healthy individuals (263). Lower levels of circulating IGF-1 were also positively correlated with a faster decline in the Mini Mental State Examination (MMSE) score in AD patients (110). Conversely, a study by Johansson et al. found that serum levels of IGF-1 and IGFBP3 were elevated in AD patients (264). The explanation for these conflicting findings is not clear, but there are many potential contributing factors. The bioactivity and bioavailability of IGF-1 are regulated by IGF-1 binding proteins (IGFBPs) (265), and the increased IGF-1 levels observed in some AD patients might be functionally suppressed by correspondingly elevated IGFBP3 levels. Support for this theory comes from a study which found a decreased ratio of IGF-1 to IGFBP3 (active/inactive) in the hippocampus of AD patients (266). Additionally, circulating IGF-1 levels change throughout the progression of AD. Several studies have reported that the early phase of AD might be associated with insulin receptor (IRs)/IGF-1R resistance, manifested as increased serum IGF-1 levels and reduced expression of IRs, IGF-1Rs, and their downstream substrates IRS-1 and IRS-2 in the brain, followed by decreased levels of serum IGF-1 at later stages of this amyloidosis (267–271).

The role of the GH/IGF-1 axis has been extensively studied in preclinical models of AD with similarly conflicting results. Many studies have suggested that IGF-1 exacerbates amyloid pathologies. Cells expressing the Swedish APP mutation treated with IGF-1 have been shown to secrete more amyloid-β peptides than untreated cells (272). *In vitro* and *in vivo* experiments demonstrated that inhibition of the IR/IGF-1 axis by NT219 (a small molecule inhibitor of scaffold proteins such as IRS1/2 that transduce IGF-1 mediated signaling) protected both cultured cells and nematodes from prion protein- or amyloid-beta-induced proteotoxicity through the formation of less toxic aggregates of higher molecular weight (273, 274). In a transgenic mouse model of AD (AβPP/PS1), treatment with picropodophyllin, a selective IGF-1R inhibitor, reduced levels of insoluble Aβ_1-40_ and Aβ_1-42_ in the temporal cortex but not in the hippocampus (275). The neuron-specific deletion of IR or IGF-1R in the mouse model of AD provided a myriad of beneficial effects, manifested by decreased APP processing, fewer amyloid plaques, less amyloid-β, improved spatial memory, and protection from premature death (276–278). Additionally, in APP/PS1 mice, GH deficiency is associated with fewer amyloid-beta plaques and lower levels of Aβ_1-40_ and Aβ_1-42_ peptides (279, 280).

However, other studies have suggested that IGF-1 may have beneficial effects in AD models. *In vitro* experiments have suggested that activating the IR/IGF-1R axis could confer protection from amyloid-β toxicity. In neuronal cultures, IGF-1 treatment decreased Aβ production and protected neurons from Aβ_25-35_- and Aβ_1-42_-induced toxicity (266, 281–284). Similarly, hippocampal overexpression of IGF-1 prevented Aβ_1-42_-induced memory loss (266). Astrocytic IGF-1 receptors prove to be crucial for the uptake of β-amyloid from neurons and the preservation of cognitive function (129). In APP/PS1 mice, treatment with IGF-1 restored levels of ADAM10; the constitutive α-secretase involved in APP processing and decreased the prevalence of Aβ_1-40_ in the cortex and hippocampus (285). However, work from other groups has suggested that enhancing GH/IGF-1 signaling either by administration of IGF-1 or by the GH secretagogue, CP-424391 failed to alter amyloid-beta clearance (286). Altogether, these observations highlight the complexity of the GH/IGF-1 axis in AD. Further studies are needed to develop a better understanding of the role of these hormones in the genesis and progression of various amyloidoses.

While development of effective anti-amyloid therapies is ongoing, physical exercise appears to improve several physiological outcomes in AD patients, including improved cognitive function, functional independence, reduced neuroinflammation and oxidative stress, and decreased cardiovascular risks (287). Evidence also shows that caloric restriction (CR) may improve cognitive performance as another beneficial lifestyle intervention (288). In both these interventions, IGF-1 levels were elevated, suggesting that the beneficial effects seen in these healthy lifestyle changes might be at least partially mediated by the GH/IGF-1 axis.

## 6 Role of GH/IGF-1 in regulation of atherogenesis

Cerebral atherogenic changes have been associated with VCID since it was first described (289), and this intracranial atherosclerosis is associated with lipid dysregulation, accumulation of cholesterol and related esters, and inflammation. Several clinical studies have found that decreased peripheral vascular health and atherosclerosis increase the risk for VCID and cognitive impairment (7, 8, 289–296). The increased risk is most robust in cases of intracranial atherosclerosis or carotid artery disease with coronary and aortic atherosclerosis having weaker or no association with VCID risk (292, 294, 297). Animal studies also support a link between atherosclerosis and VCID. When compared with control mice*, LDLr^-/-^:hApoB^+/+^* mice (a model of atherosclerosis) exhibited worsened VCID pathologies including CMH, microvascular rarefaction, BBB leakage, neuroinflammation, and cognitive impairment (298). Carotid plaque thickness and low IGF-1 levels are both independent predictors of VCID (299). Studies using aged rats demonstrated that IGF-1 supplementation reversed age-related insulin resistance, reduced serum cholesterol and triglycerides as well as reduced oxidative stress in the cortex and hippocampus (300). However, very few studies have specifically evaluated the role of IGF-1 in atherogenesis in the brain.

In contrast, there is a vast literature evaluating the role of IGF-1 and the broader somatotropic axis in systemic atherogenesis [reviewed in (231, 301–304)]. The current body of evidence suggests that IGF-1 is protective in atherosclerosis, due largely to its role in VSMCs, endothelial cells and macrophages (302, 305, 306). IGF-1 levels inversely correlate with atherosclerotic burden in a variety of animal models (307–310), as one example, systemic infusion of IGF-1 in ApoE null mice on a high-fat diet reduced vascular inflammation, reduced oxidative stress, and suppressed plaque progression (307). In part, the beneficial effects of IGF-1 in atherosclerosis have been attributed to the ability of IGF-1 to stabilize plaques *via* VSMC-mediated effects (311–313). In ApoE knockout models, supplementation of a stable IGF-1 analog stabilized plaque development by increasing vascular smooth muscle (VSMC) cell proliferation, suppressing inflammation-induced VSMC apoptosis, and increasing the cap to core ratio in early atherosclerosis. ECM regulation plays a key role in these IGF-1 mediated benefits and many of the relevant ECM proteins overlap with those important to protective IGF-1-associated cerebrovascular remodeling (discussed above) such as *Col3a1* and elastin (311). Low IGF-1 levels are also associated with increased inflammation and oxidative stress in atherosclerosis models while elevated IGF-1 is associated with improvements in those measures as well as decreased apoptosis, increased presence of VSMCs, and increased collagen (121, 307, 311, 314, 315). Studies on GH have been a little more contradictory. Overall GH is also thought to be atheroprotective based on the observation that GH deficiency is associated with premature atherosclerosis and increased prevalence of other cardiovascular risk factors (316–318). However, treatment with GH reduces only some of these risk factors, and much remains to be understood (318, 319). In addition, acromegaly patients with chronic overexpression of GH have been reported to have increased proinflammatory blood-derived cytokines, endothelial dysfunction, and increased cardiovascular mortality when compared to patients who had normal levels of GH/IGF-1 (318, 320, 321).

Recent data suggest that GH/IGF-1 may also play a role in regulating cellular senescence in the context of atherosclerosis. Senescent cells accumulate in aging, contributing to cerebrovascular pathologies and atherosclerosis (322–331). Senescent cells can cause tissue dysfunction, and IGF-1 has been shown, *in vitro*, to suppress the formation of oxidative-stress-induced senescent endothelial cells (314). Multiple types of senescent cells have deleterious effects throughout the timeline of atherosclerosis including endothelial cells, VSMCs, and macrophages/foam cells. These senescent cells contribute to disease progress and plaque rupture by promoting degradation of elastic tissue, thinning of the fibrous cap, increased plaque burden, adoption of a proinflammatory macrophage phenotype, and suppression of a protective migratory phenotype by VSMCs (329–331). Consistent with the previously discussed role of IGF-1 in promoting cap thickening and its importance as a regulator of elastic ECM components, it is not surprising that impaired IGF-1 signaling in VSMCs is important in the context of senescence-induced plaque progression. In addition to improving cap thickness, reducing lesion size, and promoting VSMC migration, treatment of high fat diet fed *Ldlr^-/-^* mice with senolytics depletes IGFBP3 in the atherosclerotic lesions (329). IGFBP3 sequesters IGF-1 preventing it from acting on the IGF-1 receptor, suggesting that one of the beneficial effects of senolytics is to increase the levels of available IGF-1 in the plaque. The importance of IGF-1 in plaques was highlighted by subsequent experiments showing that supplementation with an IGF-1 variant with reduced IGFBP3 binding or treatment with IGFBP3 neutralizing antibodies promoted adoption of the migratory VSMC phenotype which is thought to play a protective role in cap maintenance and repair (329). Treatment with GH, and the subsequent increases in IGF-1 have also been shown to decrease the number of senescent endothelial progenitor cells, a key part of the vascular repair process in atherosclerosis (332).

## 7 Sex-based differences in the GH/IGF-1 axis in the aging cerebrovasculature

Sexual dimorphism and sex-based differences in phenotypes associated with the GH/IGF-1 signaling axis are complex (333, 334). There is evidence for complicated interactions between the somatotropic and gonadotropic axes during development and during pubertal growth (334, 335). In the context of aging, studies in Ames dwarf mice (336) showed that both females and males had increased longevity compared to controls. GH receptor knockout mice also exhibited increased longevity in both females and males, although there was variation across genetic backgrounds (337). In contrast, increased longevity in *Igf1r^+/-^* mice was seen only in females (143, 338). The mechanisms underlying these sex differences are not known.

There has been some work evaluating sex-differences associated with the GH/IGF-1 axis in the cardiovascular system and brain. Several sex-specific differences in cardiac function are eliminated in liver specific IGF-1 knockouts. This has been attributed to the fact that female C57BL/6 mice have higher circulating levels of IGF-1 than male mice but the liver-specific knockout reduces IGF-1 to low levels that are similar in both males and females (339). Estrogen and IGF-1 can both exert neuroprotective effects, and there have been reports suggesting that the two pathways can act cooperatively in the context of ischemic stroke (252, 253, 335, 340). In addition, activation of estrogen (E_2_) receptors has been shown to increase IGF-1 uptake into brain endothelial cells, resulting in some sex-specific differences in IGF-1 bioavailability (341).

More recent work has demonstrated that post-developmental neuronal over-expression of IGF-1 rescued age-related defects in neuromuscular function in males but not females (105). Chronic overexpression of IGF-1 did not ameliorate age-related losses in cognitive function, although short-term delivery (4-weeks intranasal) of IGF-1 in aged male mice did lead to minor improvements in cognitive function (female mice were not evaluated) (105). However in most studies evaluating the effects of IGF-1 deficiency on the vasculature, including those studying neurovascular coupling, blood-brain-barrier permeability, development of CMH, vascular structure and function, progression of atherosclerosis, etc. sex-specific effects were either not evaluated or not reported, making this an area ripe for further exploration.

## 8 Conclusion and perspectives

There is a strong body of evidence highlighting the vasoprotective effects of IGF-1, particularly in the cerebral vasculature. IGF-1 is essential for brain vascular health, and IGF-1 deficiency in aging contributes to the development of VCID and cognitive impairment. It can be difficult to separate out the effects of GH from those of IGF-1, since one of the primary functions of GH is to induce secretion of IGF-1. However, there are also direct effects of GH in some systems which may be tied to effects of GH signaling on NO production (342). While much is known about GH/IGF-1 axis in aging and cardiovascular disease, much remains to be explored. In particular, the cellular contributions to various pathologies remain incompletely understood. It can also be hard to dissect out what the contributions of GH/IGF-1 signaling on individual cell types in complicated multicellular pathologies such as atherogenesis and cerebrovascular dysfunction. Part of the challenge lies in the fact that so many of the cells involved (endothelial cells, astrocytes, neurons, VSMCs, macrophages) can all respond to IGF-1 and contribute to disease development. In addition, while GH and IGF-1 are most often thought of as circulating systemic factors that act in a cell non-autonomous way, there is locally produced IGF-1 in many tissues, including the brain and atherosclerotic plaque. Dissecting the contributions of these different IGF-1 pools can be time-consuming and challenging with current animal models. Much remains to be explored regarding the downstream molecular mediators of GH/IGF-1 vasoprotective effects. Canonical signaling through GH/IGF-1 has been well-established for decades but it remains unclear whether there are tissue specific downstream mediators that might make good therapeutic targets without the broader effects of delivering hormones with as many pleiotropic effects as GH/IGF-1.

## Author contributions

All authors listed have made a substantial, direct, and intellectual contribution to the work and approved it for publication.
